# OralSegNet: An Approach to Early Detection of Oral Disease Using Transfer Learning

**DOI:** 10.1111/odi.70135

**Published:** 2025-11-09

**Authors:** Pranta Barua, Md Rakibul Islam, Mahmud Uz Zaman, Adel Alenazi, Fawaz Alqahtani, Huda Abutayyem, Maher Al Shayeb, Ruba Odeh, Nesrine A. Elsahn, Mohammad Khursheed Alam

**Affiliations:** ^1^ Department of Computer Science and Engineering University of Chittagong Chittagong Bangladesh; ^2^ Department of Computing Universiti Teknologi PETRONAS (UTP) Perak Darul Ridzuan Malaysia; ^3^ Department of Software Engineering Daffodil International University Dhaka Bangladesh; ^4^ Department of Oral and Maxillofacial Surgery and Diagnostic Sciences, College of Dentistry Prince Sattam Bin Abdullaziz University Al‐kharj Saudi Arabia; ^5^ Department of Prosthetic Dental Sciences, College of Dentistry Prince Sattam Bin Abdullaziz University Al‐kharj Saudi Arabia; ^6^ Department of Clinical Sciences, Center of Medical and Bio‐Allied Health Sciences Research, College of Dentistry Ajman University Ajman UAE; ^7^ Preventive Dentistry Department, College of Dentistry Jouf University Sakaka Saudi Arabia; ^8^ Department of Dental Research Cell, Saveetha Institute of Medical and Technical Sciences, Saveetha Dental College and Hospitals Chennai India; ^9^ Department of Public Health, Faculty of Allied Health Sciences Daffodil International University Dhaka Bangladesh

**Keywords:** AI, caries, deep learning, gingivitis, instance segmentation, intraoral images, mouth ulcer, ONNX deployment, periodontitis, YOLOv11

## Abstract

**Objective:**

Deep learning‐based segmentation system is proposed that exploits three variants of YOLOv11 architecture, namely YOLOv11n‐seg, YOLOv11s‐seg, and YOLOv11m‐seg for automated detection and localization of the oral disease conditions from photographic intraoral images.

**Method:**

Dataset has been created by combining publicly available data from sources like Roboflow resulting in an initial version (v1) having 582 images annotated at the pixel level. To mitigate class imbalance issues as well as increase generalization capability by the model, progressively this dataset was augmented to create version 2 (v2) and then further extended into version 3 (v3). Training was done in three separate stages: Feature extraction, partial fine‐tuning, and full fine‐tuning.

**Results:**

YOLOv11m‐seg model performed best in the partial fine‐tuning phase with results of box mAP@50 = 0.521 and mask mAP@50 = 0.500. Training was done on Google Colab's free tier using an Intel Xeon CPU with 13 GB RAM, 15 GB T4 GPU, and 120 GB storage allowance.

**Conclusions:**

For application, the best performing model was exported to ONNX format with NMS enabled and deployed as a fully client‐side responsive web app built in React.js and ONNX Runtime Web. The tool enables both clinicians and non‐experts to detect oral diseases from intraoral images with a single click.

## 1 Introduction

Oral diseases (dental caries, gingivitis, periodontitis and mouth ulcers) affect billions of people worldwide, and still rank among the most prevalent non‐communicable diseases. Early diagnosis is fundamental because untreated caries can progress to pulpitis and loss of the tooth, and gingivitis and periodontitis are major factors of dental morbidity in adults and have been associated with other health problems such as cardiovascular diseases and diabetes. However, normal clinical examination and radiographic imaging are subjective to skilled personnel and may miss small and early lesions, especially in under‐resourced regions. Manual assessments are also time‐intensive and prone to inter‐observer variability, highlighting the urgent need for automated and reliable screening tools. The latest advances in deep learning (DL) have changed medical image analysis dramatically, allowing models to learn intricate feature hierarchies from data, sparing the handcrafting of features and manual tuning (Zhang et al. 2023). In particular, convolutional neural networks (CNNs) have shown outstanding performance for dental diagnostics. Chau et al. (2023) used DeepLabv3+ with Xception and MobileNetV2 to detect gingivitis from intraoral images, obtaining sensitivity 0.92, specificity 0.94, and mean IoU 0.60. Hsung and coworkers proposed a mobile side DeepLabv3+ (Xception65 and MobileNetV2) for gum disease detection and achieved a comparable IoU on a smaller intraoral set (Cheng et al. 2024). Fatima et al. (2023) combined MobileNetV2 and Mask R‐CNN for multiclass dental lesion segmentation, reporting 94% accuracy, mAP 0.85, and IoU 0.71. Zhu et al. (2023) introduced CariesNet a U‐shaped network with axial attention for multi‐stage caries segmentation on 1159 panoramic radiographs, achieving Dice 0.9364 and accuracy 0.9361. Park et al. (2022) improved intraoral caries classification from 75.8% to 81.3% using a hybrid of U‐Net, ResNet‐18, and Faster R‐CNN, after segmenting tooth surfaces. Pediatric benchmarks on mixed adult/child panoramic reached IoU 0.8389, recall 0.92, and accuracy 0.9710 (Zhang et al. 2023), while high‐order focus models achieved Dice 0.7972 for oral ulcer detection (Jiang et al. 2024). Despite these advances, most existing research targets a single condition or specific imaging modality, which limits utility in broader, point‐of‐care settings (Park et al. 2022). Instance segmentation models such as Mask R‐CNN and U‐Net deliver pixel‐level precision but usually require multi‐stage pipelines and heavy computation (Fatima et al. 2023; Alsolamy et al. 2025). Related studies further emphasize the clinical and biological dimensions of oral health research. Enezei et al. (2020) analyzed mandibular fracture patterns and outcomes, highlighting the importance of accurate diagnostic assessment, while Enezei et al. (2017) demonstrated enhanced dental stem cell regeneration on bioactive scaffolds. By contrast, the YOLO series is optimized for real‐time object detection, and its latest versions (YOLOv8‐Seg and YOLOv11‐Seg) now support integrated mask prediction in a single forward pass. Transfer learning using pretrained backbones remains a powerful tool to overcome data scarcity in oral health applications (Zhang et al. 2023), but systematic evaluations of YOLOv11‐Seg for dental disease detection are still lacking.

To address these gaps, we present OralSegNet, a transfer learning‐based segmentation framework that employs three YOLOv11‐Seg variants (nano, small, and medium) to detect and segment four common oral diseases from standard intraoral photographs. We compiled a pixel‐level annotated dataset of 582 images (v1) and expanded it with targeted augmentations to create more balanced and generalizable v2 and v3 datasets (Roboflow Universe 2025). Each model was trained in three phases: feature extraction, partial fine‐tuning, and full fine‐tuning, while passing each phase's trained learning to the next phase, in order to balance model complexity against overfitting risk. The best performer was exported to ONNX and deployed entirely client‐side via ONNX Runtime Web, enabling real‐time, secure inference within a lightweight React.js interface. By unifying detection and segmentation in a single step, OralSegNet supports fast, scalable early oral disease screening with strong potential to improve clinical workflows and extend preventive dental care to underserved populations.

## 2 Literature Review/Previous Works Study

Chau et al. (2023) used DeepLabv3+ with Xception and MobileNetV2 backbones for gingivitis detection in 567 frontal view intraoral images. They achieved sensitivity of 0.92, specificity of 0.94, and mean IoU of 0.60; however, results can hardly generalize beyond their Chinese‐only dataset. Cheng et al. (2024) also used DeepLabv3+, specifying Xception65 and MobileNetV2, for mobile‐based gum disease detection; they reported mean IoU 0.60 on a small intraoral set an indicator that there might be robustness issues. Fatima et al. (2023) merged a lightweight MobileNet‐V2 backbone with Mask R‐CNN to segment multiclass dental lesions with accuracy = 94%, and mAP = 0.85, and IoU = 0.71 all on somewhat limited datasets. Zhu et al. (2023) proposed CariesNet a U‐shaped network with axial attention on 1159 panoramic X‐rays, achieving Dice 0.9364 and accuracy 0.9361 for multi‐stage caries. However, results based on panoramics are not very relevant to the intraoral photos used here. Park et al. (2022) employed U‐Net, ResNet‐18, and Faster R‐CNN on intraoral images. They increased AUC from 0.731 to 0.837 with segmentation, but it required high‐quality annotations. Zhang et al. (2023) released a children's panoramic dataset (584 images) and evaluated U‐Net, R2 U‐Net, PSPNet, and DeepLabV3 reaching IoU 0.8389, recall 0.92, and accuracy 0.9710 though mixing child and adult data introduced variability. Jiang et al. (2024) designed HF‐UNet for ulcer segmentation on a diverse ulcer dataset, achieving Dice 0.7972, accuracy 0.9715, specificity 0.9932, and sensitivity 0.7257, with performance tied to annotation quality. He et al. (2024) trained YOLOv11‐seg on high resolution (1280 × 1280) construction images for 1000 epochs, reaching mAP@0.5 0.808; this work's segmentation insights inform dental adaptations. Alsolamy et al. (2025) fine‐tuned YOLO‐based detection on 584 bitewing radiographs for proximal caries, reporting precision 0.844, recall 0.864, F1 0.851, and mAP 0.888. Tareq et al. (2023) built a hybrid YOLOv5 ensemble on 1703 smartphone caries images, achieving mAP 0.732, accuracy 0.789, and recall 0.701; a transfer learned VGG16 then reached 86.96% accuracy, precision 0.89, and recall 0.88. Bayati et al. (2025) used YOLOv8 on 1506 bitewings, securing precision 0.9603 (enamel) and 0.8006 (dentin), with overall precision 0.8483, recall 0.7977, and F1 0.8222. Hua et al. (2025) introduced YOLO‐DentSeg (YOLOv8n‐seg with C2f‐Faster, BiFPN, and attention) on 2720 panoramic images, achieving mAP50 (box) 0.87 and mAP50 (mask) 0.855 at 90 FPS, reducing model size by ~45%. Ramírez‐Pedraza et al. (2025) employed YOLOv11m to detect three plaque stages in 2000 intraoral photos, reporting mAP@0.5 0.713; lighter plaque (new) remained challenging under variable lighting. Wen et al. (2024) developed a DenseNet‐based CNN for grading gingival inflammation on intraoral images, attaining mean IoU 0.727 and 74%–79% classification accuracy across inflammation levels, with performance tied to image quality. Zhou et al. (2024) evaluated YOLOv5 for aphthous ulcer detection on clinical photos, achieving precision 0.987 and recall 0.795 (F1 0.881), indicating strong localization but limited recall for subtle ulcers. Chen et al. (2023) combined YOLOv7 (tooth localization) and EfficientNet‐B0 (per tooth classification) on 1200 periapical radiographs, yielding YOLOv7 AP 0.971 for detection and EfficientNet AUC 0.9867 (periodontitis) and 0.9831 (caries). Li et al. (2021) implemented a CNN classifier on 2000 intraoral photos for gingivitis screening with > 95% accuracy (specific metrics were not consistently reported). Park et al. (2023) trained a custom CNN on 1500 color tooth images to classify periodontal disease, achieving ~91% accuracy via smartphone‐captured data. Finally, Jiang et al. (2022) combined U‐Net and YOLOv4 for staging periodontal bone loss on 1000 panoramic images: YOLOv4 detected landmarks with precision 0.88 (recall 0.51) and U‐Net segmented bone loss, yielding overall accuracy ~0.85 despite moderate recall. Collectively, these studies confirm that transfer learned CNNs and YOLO variants can deliver high performance segmentation and detection across diverse oral imaging tasks.

Although previous studies achieved strong performance on specific oral imaging tasks, most exhibited important limitations. For instance, Fatima et al. (2023) and Zhu et al. (2023) employed comparatively small or modality‐restricted datasets (panoramic radiographs rather than intraoral photos), while architectures like Mask R‐CNN, U‐Net, and DeepLabV3+ demanded high computational costs and multi‐stage pipelines. Larger‐scale studies such as Hua et al. (2025) (2720 images) and Ramírez‐Pedraza et al. (2025) (2000 images) used heavier or specialized YOLO variants, trading real‐time performance for complexity.

Despite varied tasks and datasets, the literature consistently shows that transfer learning with deep CNNs yields excellent performance in early oral disease detection. Multiple authors highlight that leveraging pretrained models such as (e.g., VGG, ResNet, Inception) on oral image data greatly improves accuracy and convergence. In summary, current research demonstrates that advanced CNN architectures, especially when fine‐tuned or combined with traditional image processing, can reliably identify a wide range of oral pathologies (gingivitis, periodontitis, caries, oral cancer, etc.) from photographic and radiographic images. These findings provide a strong foundation for our research to develop an automated, transfer learning‐based system for early detection of oral disease. Our study focuses on a unified, lightweight YOLOv11‐Seg framework trained on a balanced, pixel‐annotated intraoral dataset that emphasizes efficiency, cross‐condition detection, and browser‐side deployment. This tries to address the prevailing gap between high‐accuracy research models and clinically practical, low‐resource solutions.

## 3 Methodology

**FIGURE 1:**
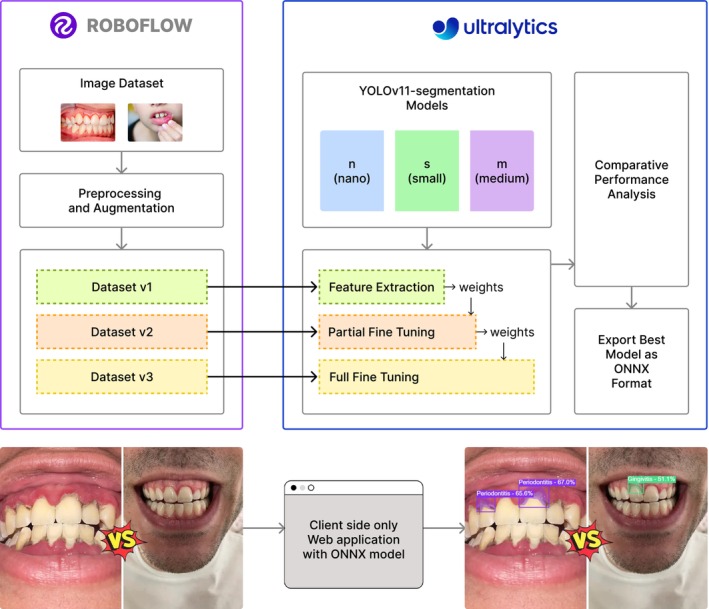
Block diagram of system architecture.

The proposed system is designed to automatically segment and classify four common dental diseases such as dental caries, gingivitis, mouth ulcers, and periodontitis from intraoral images shown in Figure 1. The system employs state‐of‐the‐art YOLOv11‐seg models in three variants (n, s, m) provided by the Ultralytics (Jocher et al. 2024) platform, using a multi‐phase training strategy that includes feature extraction, partial fine‐tuning, and full fine‐tuning.

### 3.1 Overview of YOLOv11‐Segmentation Model

Released by the Ultralytics team on 30 September 2024, YOLOv11 represents the latest breakthrough in real‐time object detection technology. This version supports various tasks, such as detecting objects, segmenting images, classifying images, estimating poses, and detecting objects with specific orientations. Each YOLOv11‐seg model consists of three major components: backbone, neck, and head.

#### 3.1.1 Backbone

Extracts multi scale features from input images using the following modules:

- *Conv*: Initial layer that extracts basic image features.
- *C3k2*: Combines convolutions and skip connections for efficient local feature extraction.
- *SPPF*: Uses pooling at different scales to capture global context.
- *C2PSA*: Enhances important features and filters noise for better detection in complex scenes.

#### 3.1.2 Neck

Connects the backbone and head, merging multi‐scale features to help detect objects of different sizes. It includes:

- *Upsample*: Increases resolution to better detect small objects.
- *Concat*: Merges features from different layers to combine detailed information.
- *C3k2*: Enhances features through repeated convolutions and shortcut paths for better accuracy.

#### 3.1.3 Head

The head converts features into final detection results. It uses the detect and segment module to process features at multiple scales, helping accurately detect and segment both large and small objects. This stage ensures precise object recognition across varied sizes (Table 1).

**TABLE 1:** Layer and module count of YOLOv11‐segmentation models.

Variant	Backbone modules	Backbone layers	Neck modules	Neck layers	Head modules	Head layers	Total layers
n (nano)	11	36	12	28	1	139	203
s (small)	11	36	12	28	1	139	203
m (medium)	11	36	12	28	1	189	253
l (large)	11	41	12	30	1	308	379
x (x‐large)	11	41	12	30	1	308	379

On the COCO dataset (n, s, m) variants of the model got below results shown in Table 2.

**TABLE 2:** Results of YOLOv11‐segmentation (n, s and m) model on the COCO dataset.

Model	Size (pixel)	mAP^box^ 50–95	mAP^mask^ 50–95	Params (M)	FLOPs (B)
YOLOv11‐n	640	38.9	32.0	2.9	10.4
YOLOv11‐s	640	46.6	37.8	10.1	35.5
YOLOv11‐m	640	51.5	41.5	22.4	123.3

### 3.2 Dataset

#### 3.2.1 Dataset Splitting and Balancing Annotations

**FIGURE 2:**
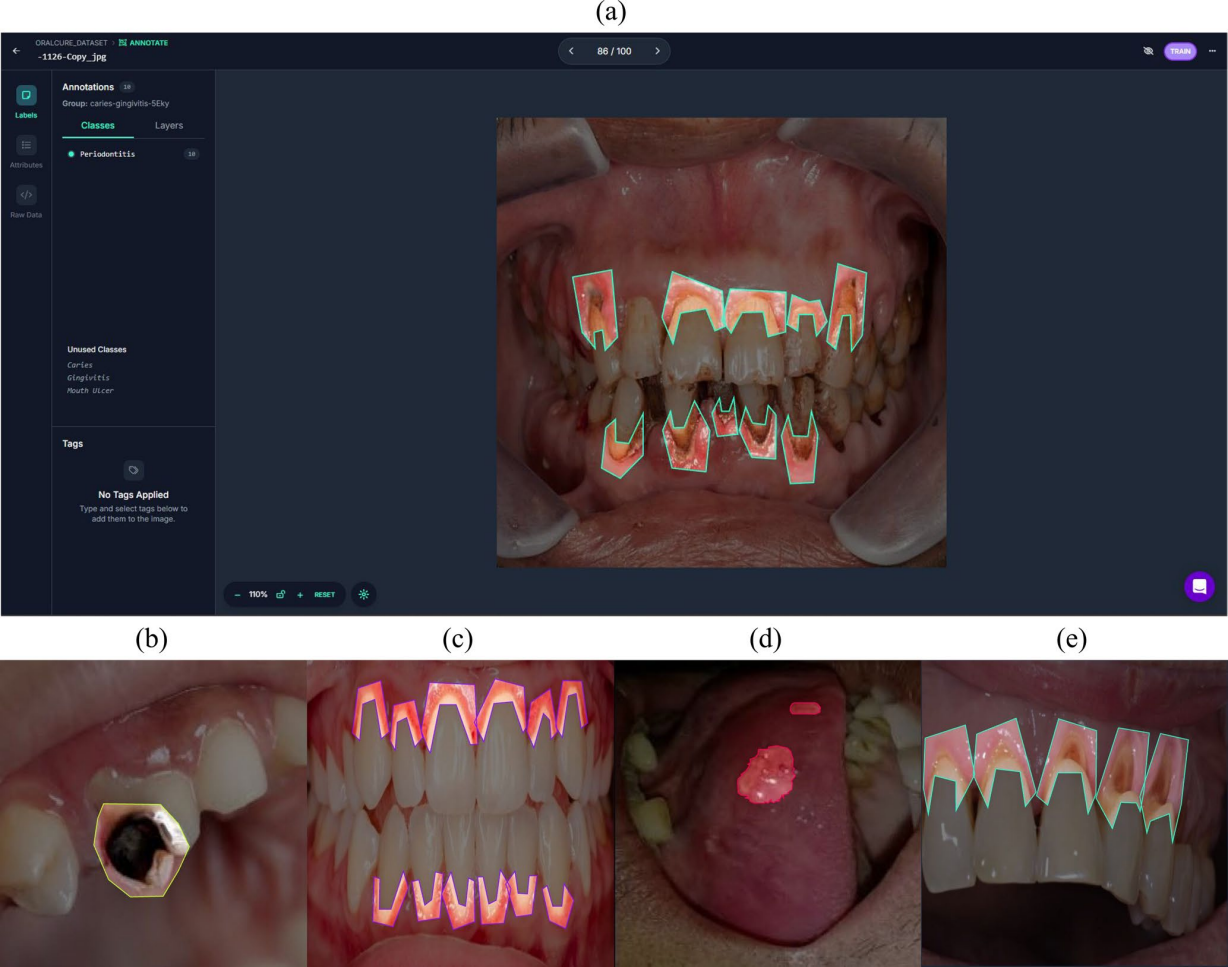
(a) Dataset annotation using the Roboflow platform; following are the annotations of (b) 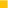 caries, (c) 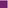 gingivitis, (d) 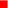 mouth ulcer and (e) 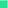 periodontitis.

The initial dataset of 582 images was split into train, validation and test, where there were 429 train, 77 validation and 76 test images. To keep the annotation, count the same for each class, a manually balanced instance/annotation count was used for the train and validation split. The train annotation count was 300 for each class, and the validation annotation count was 46 for each class. Later after applying augmentation the annotation count of the training split increased proportionally, while the validation and test splits stayed the same, ensuring no data overlap to prevent leakage (Figure 2).

#### 3.2.2 Dataset Preprocessing and Augmentation

To increase the variety and ability to generalize of the model, preprocessing and augmentation methods were done only to the training part of the data set. As a prep step, all images were resized in a standard way to 640 × 640 pixels by stretching them to match input size dimensions of the model. For augmentation, each train image was made into three different versions using a mix of ops. Sideways flipping was used to show left–right similarity, while random turns from −5° to +5° added slight angle changes. Warping was done both sideways and up down within ±3°, resembling small distortions in camera view. Brightness adjustments were made in the range of −10% to +10%, alongside exposure variations from −5% to +5%, to simulate lighting inconsistencies. Furthermore, a mild Gaussian blur of up to 1.5 pixels was used to replicate slight focus shifts, and random noise affecting up to 1% of the pixels was added to account for sensor‐level imperfections. These transformations collectively increased the robustness of the model against real‐world variations in intraoral image quality.

#### 3.2.3 Dataset Versioning

To support multi‐phase training, we created three versions of our dataset shown in Table 3. We applied augmentation on train split of V1 to get V2, and very mild augmentation (Rotation ±2°, Brightness ±5°, Noise 0.5%) on train split of V2 to get V3. Which increases image and annotation count 3× for train split only.

**TABLE 3:** Dataset versioning.

Version	Train image count	Train annotation count per class	Total train annotation count	Validation image count	Validation annotation count per class	Test image count	Test annotation count
V1	429	300	1200	77	46	76	Untouched
V2	1287	900	3600	77	46	76	Untouched
V3	3861	2700	10,800	77	46	76	Untouched

### 3.3 Multi Phase Training

To support transfer learning, we employed a layered approach which consists of feature extraction, partial fine‐tuning, and full fine‐tuning.

- *Feature extraction*: The model's detection head is trained while keeping the backbone frozen, allowing for rapid adaptation to the dental domain.
- *Partial fine tuning*: A subset of network layers is fine‐tuned, with only the first five layers remaining frozen. This enables deeper layers to adapt while preserving critical low‐level features.
- *Full fine tuning*: A larger model variant undergoes full fine tuning (all layers unfrozen) over an extended 200 epoch training run. This phase refines the network's ability to accurately segment and classify subtle and challenging conditions, such as gingivitis and periodontitis.

Training hyperparameters for all three variants (n, s, m) of the YOLOv11‐seg model are shown in Table 4.

**TABLE 4:** Training parameters.

Parameter	Feature extraction	Partial fine tuning	Full fine tuning	Description
Dataset	V1	V2	V3	Dataset version with increasing augmentation
Freeze	11	5	None	Number of layers to freeze. 11 freezes entire backbone, 5 freezes half
Learning rate	0.01	0.001	0.005	Decreasing learning rate for finer adjustments as more layers are unfrozen
Epochs	50	100	200	Number of training epochs, monitored for convergence
Batch size	32	32	32	Fixed batch size, ensuring hardware compatibility
Image size	640	640	640	Default size by Ultralytics
Cosine LR	True	True	True	Enables cosine learning rate scheduler for better convergence
Optimizer	AdamW	AdamW	AdamW	AdamW extends Adam, adding weight decay to improve generalization, optimizing gradients for faster convergence
Patience	20	20	20	Epochs to wait without validation improvement before early stopping
Warmup_epochs	N/A	0	0	Stablize the training while continuing from last epoch

*Note:* Ultralytics does provide many default parameters for training. The above parameters were manually updated and rest kept same. Default train parameters are found at: https://docs.ultralytics.com/modes/train/#train‐settings.

### 3.4 Model Deployment

We deploy our trained YOLOv11‐Seg model using the Open Neural Network Exchange (*ONNX*) format an open standard that defines a unified computation graph and operator set. By exporting the model with its built‐in non‐maximum suppression (NMS) to ONNX, we gain broad compatibility across platforms and eliminate the need for a dedicated Python server.

On the client side, we use *ONNX Runtime Web*, a lightweight JavaScript library that runs ONNX models directly in the browser using *WebAssembly*, *WebGL*, or *WebGPU*. This setup offers several major advantages:

- *No backend required*: All computation takes place locally on the user's machine, so there is no need for external servers. This greatly eases deployment and upkeep.
- *Real time*: Running the model in‐browser removes network delays, enabling fast real‐time predictions.
- *Cross‐platform support*: ONNX Runtime Web hides hardware differences, letting the same ONNX model run the same across desktops, tablets, and smartphones whether they use CPUs or GPUs.

React.js is used for building frontend and loading the ONNX model into the browser at startup. Components of React call ONNX Runtime Web APIs to run inference and display detection results in real time. The fully client‐side pipeline delivers a fast, seamless user experience with no server‐side infrastructure needed.

### 3.5 Evaluation Metrics

To assess the effectiveness of the YOLOv11‐Segmentation multi‐phase training framework, performance is evaluated on both object detection (bounding boxes) and segmentation (masks) using standard metrics. In addition, loss metrics are monitored during training. For clarity and consistency, common formulas used across multiple metrics are defined first and then referenced throughout the evaluation.

#### 3.5.1 Common Metrics

##### 3.5.1.1 Precision (*P*)

$$P=\frac{\mathrm{TP}}{\mathrm{TP}+\mathrm{FP}}$$

where TP is the number of true positives and FP is the number of false positives.

##### 3.5.1.2 Recall (*R*)

$$R=\frac{\mathrm{TP}}{\mathrm{TP}+\mathrm{FN}}$$

where FN is the number of false negatives.

##### 3.5.1.3 Intersection Over Union (IoU)

For a given predicted region and the corresponding ground truth, the IoU is calculated as:

$$\mathrm{IoU}=\frac{\text{Area of Overlap}}{\text{Area of Union}}=\frac{\sum_{i=1}^{N}\left(y_{i}^{\text{pred}}\cdot y_{i}^{\text{true}}\right)}{\sum_{i=1}^{N}\left(y_{i}^{\text{pred}}+y_{i}^{\text{true}}-y_{i}^{\text{pred}}\cdot y_{i}^{\text{true}}\right)}$$

where $y_{i}^{\text{pred}}$ and $y_{i}^{\text{true}}$ are binary (or thresholded) pixel values and *N* is the total number of pixels.

##### 3.5.1.4 Average Precision (AP)

For each class, AP is computed as the area under the precision–recall curve:

$$\mathrm{AP}=\int_{0}^{1}P\left(R\right)\;\mathrm{dR}$$

##### 3.5.1.5 Mean Average Precision (mAP)

Averaged over all number of classes:

$$\mathrm{mAP}=\frac{1}{N_{\text{classes}}}\sum_{c=1}^{N_{\text{classes}}}{\mathrm{AP}}_{c}$$

Evaluation is performed at a fixed IoU threshold which is 0.5 (e.g., mAP@50) or across a range (e.g., mAP@50–95).

#### 3.5.2 Bounding Box Metrics

These metrics evaluate how accurately the model localizes dental lesions with bounding boxes.

##### 3.5.2.1 Precision and Recall

As defined above, these measure the ratio of correct detections versus total detections and the ability to detect all true instances, respectively.

##### 3.5.2.2 mAP@50 and mAP@50–95

For bounding boxes, a detection is considered correct if the IoU between the predicted and the ground truth bounding box is at least 0.5 (for mAP@50) or across multiple thresholds (for mAP@50–95). The AP for each class is computed as the area under its precision–recall curve and then averaged to yield mAP.

#### 3.5.3 Mask Metrics

For segmentation performance, the metrics focus on pixel‐level accuracy of the predicted masks.

##### 3.5.3.1 Pixel‐Wise Precision and Recall

Calculated in the same manner as for bounding boxes, but using pixel level predictions:

$$P_{\text{mask}}=\frac{TP_{\text{mask}}}{TP_{\text{mask}}+FP_{\text{mask}}},\;R_{\text{mask}}=\frac{TP_{\text{mask}}}{TP_{\text{mask}}+FN_{\text{mask}}}$$

##### 3.5.3.2 mAP@50 and mAP@50–95 for Masks

Similar to bounding box metrics, these are computed by evaluating the IoU between the predicted mask and the ground truth mask, where the AP is averaged over all classes.

#### 3.5.4 Loss Metrics

During training, several loss functions are combined and provided to guide learning.

##### 3.5.4.1 Box Loss (*L*_box_)

Typically computed using smooth L1 or GIoU loss to measure discrepancies between predicted and ground truth bounding boxes:

$$\begin{array}{c} L_{\mathrm{box}}=\lambda_{\text{coord}}\sum_{i=1}^{N}[\text{Smooth}L1\left(x_{i}-x_{i}^{*}\right)+\text{Smooth}L1\left(y_{i}-y_{i}^{*}\right)\\ \;+\text{Smooth}L1\left(w_{i}-w_{i}^{*}\right)+\text{Smooth}L1\left(h_{i}-h_{i}^{*}\right)] \end{array}$$

which can be simplified to:

$$L_{\mathrm{box}}=\lambda_{\text{coord}}\sum_{i=1}^{N\text{Smooth}L1}\left(\mathrm{bbo}x_{i}^{\text{pred}}-b{\mathrm{box}}_{i}^{\text{true}}\right)$$

where *x*, *y* = predicted box center coordinates, *w*, *h* = predicted width and height, x*, y*, w*, h* = ground‐truth box parameters, $\lambda_{\text{coord}}$ = weight controlling localization importance, and SmoothL1 = a loss function that combines L1 loss with a smooth transition to reduce the influence of outliers on the overall loss.

##### 3.5.4.2 Segmentation Loss (*L*_seg_)

A combination of cross‐entropy loss and dice loss, which is particularly important for capturing fine object boundaries:

$$L_{\mathrm{seg}}=\lambda_{\mathrm{CE}}\cdot \text{CrossEntropy}\left(P,T\right)+\lambda_{\text{Dice}}\cdot \left(1-\frac{2\sum \left(P\cdot T\right)}{\sum P+\sum T}\right)$$

where *P* = predicted mask,*T* = ground truth mask, $\lambda_{\mathrm{CE}}$ and $\lambda_{\text{Dice}}$ = weights balancing pixel‐level and region‐level accuracy.

##### 3.5.4.3 Classification Loss (*L*_cls_)

Evaluated using the cross‐entropy loss:

$$L_{\mathrm{cls}}=-\frac{1}{N}\sum_{i=1}^{N}\sum_{c=1}^{C}y_{i,c}\log\left(p_{i,c}\right)$$

where $y_{i,c}$ = ground truth indicator for class c of sample i., $p_{i,c}$ = predicted probability for class c, *N* = number of samples, and *C* = number of classes.

##### 3.5.4.4 Distribution Focal Loss (*L*_DFL_)

Improves the model's focus on difficult to classify samples by refining the probability distributions:

$$L_{\mathrm{DFL}}=-\sum_{i=1}^{N}y_{i}\log\left(p_{i}\right)$$

where *y_i_* = target distribution, *p_i_* = predicted distribution.

The overall loss is a weighted sum of these components:

$$L_{\text{total}}=L_{\mathrm{box}}+L_{\mathrm{seg}}+L_{\mathrm{cls}}+L_{\mathrm{DFL}}$$

## 4 Result and Analysis

The system is evaluated using standard segmentation performance metrics like precision, recall, mAP@50 and mAP@50–95 for both detection box and segmentation of the object. First, we start with analyzing the performance of individual models across phases of training for all classes, shown in Table 5

**TABLE 5:** Result of YOLOv11‐seg (n, s and m) models.

Model	Phase	Box precision	Box recall	Box mAP@50	Box mAP@50–95	Mask precision	Mask recall	Mask mAP@50	Mask mAP@50–95
YOLOv11n‐seg	FE	0.519	0.564	0.483	0.263	0.511	0.541	0.474	0.226
	PFT	0.549	0.535	0.523	0.290	0.544	0.529	0.510	0.246
	FFT	0.519	0.564	0.483	0.263	0.511	0.541	0.474	0.226
YOLOv11s‐seg	FE	0.479	0.587	0.487	0.261	0.474	0.581	0.481	0.221
	PFT	0.440	0.609	0.493	0.282	0.565	0.484	0.490	0.243
	FFT	0.491	0.543	0.488	0.258	0.550	0.489	0.481	0.221
**YOLOv11m‐seg**	FE	0.491	0.462	0.435	0.256	0.501	0.466	0.445	0.224
	**PFT**	**0.574**	**0.566**	**0.521**	**0.277**	**0.560**	**0.553**	**0.500**	**0.228**
	FFT	0.545	0.451	0.472	0.246	0.541	0.429	0.453	0.187

*Note:* The bold formatted text YOLO11m‐seg model at partial fine‐tuning phase achieved the highest metrices among all thus the final result/outcome of the experiment.

We observed that the YOLOv11m‐seg model with partial fine tuning (PFT) delivered the strongest overall performance across all configurations tested. It achieved the highest values in both Box metrics (Precision: 0.574, Recall: 0.566, mAP@50: 0.521, mAP@50–95: 0.277) and Mask metrics (Precision: 0.560, Recall: 0.553, mAP@50: 0.500, mAP@50–95: 0.228). These results suggest that the medium scale model strikes a strong balance between capacity and generalization when moderately fine‐tuned. Notably, the n‐seg model showed gains during partial fine tuning compared to its feature extraction (FE) phase, but performance plateaued or even declined slightly with full fine tuning (FFT). This could indicate overfitting or that the smaller model had already reached its capacity limit. The s‐seg model in mask related metrics benefited from partial fine tuning, but saw a modest drop in performance during full fine tuning. This illustrates how extended training might begin to wear away its ability to generalize. Taken together, these findings highlight the value of partial fine tuning, which appears to be able to take advantage of pre‐trained weights without overfitting. In all of the tested setups, YOLOv11m‐seg with partial fine tuning emerged as a very robust and balanced model. The results underscore how model scaling and phased training strategies optimize transfer learning architectures by utilizing model's pretrained features, specifically for the clinical segmentation of dental diseases.

**FIGURE 3:**
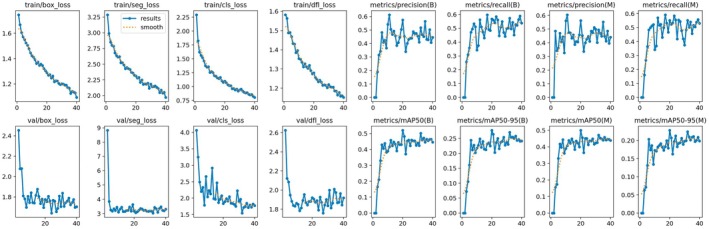
Result of best performing model observed on YOLOv11m‐seg partial fine‐tuning phase, where (B) = Box, (M) = Mask.

Figure 3 presents the training dynamics of the YOLOv11m‐seg model in its partial fine‐tuning phase. Shown in the top row of plots, is a steady decrease in the training losses of box loss, segmentation loss, classification loss, and distribution focal loss (DFL) as epochs progress. Such a steady downward trend implies that the model is effectively learning meaningful patterns from the available training data.

The second row shows the validation losses, which continue in much the same way after pulling back initially. The marked drop in segmentation and classification losses hints that the model is not just overfitting to the training set but is rather generalizing well to new, unseen data. The loss curves have been very smoothly taken, with only minor fluctuations, to reflect a stable and well‐controlled training process. As for the performance metrics, the plots show an absolutely steady improvement in precision and recall for both bounding box (B) and mask (M) predictions. And notably, both mAP@50 and mAP@50–95 values are increasing over time, with the mask metrics displaying slightly better consistency and less variation. These trends confirm that as training continues, the model becomes more precise in both detecting and outlining the diseased areas on teeth underscoring how well the partial fine‐tuning approach works.

**FIGURE 4:**
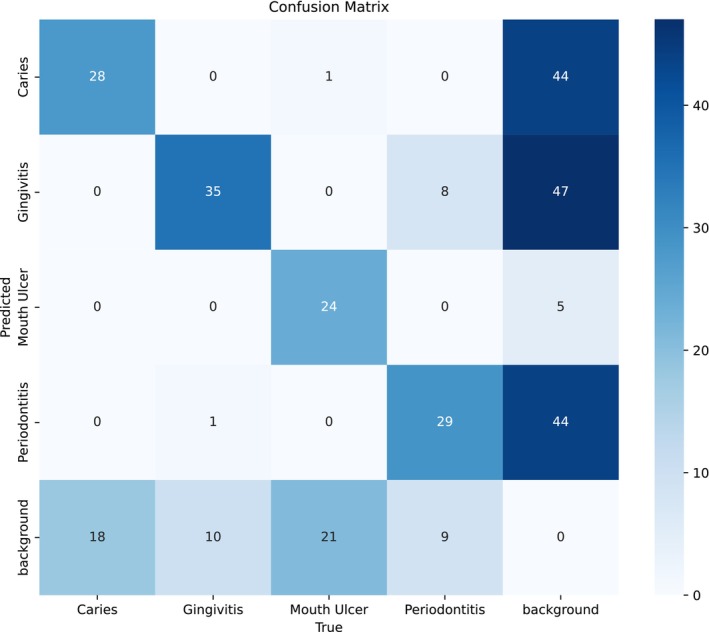
Confusion matrix of best performing model observed on YOLOv11m‐seg partial fine‐tuning phase.

Shown in Figure 4 is a confusion matrix for the model's predictions over five classes. The diagonal elements are correct classifications; it has exhibited very good performance for Gingivitis and Periodontitis which is very important to this experiment since these two classes have very little difference; they are distinctly related.

### 4.1 Disease Detection Using Web Application

**FIGURE 5:**
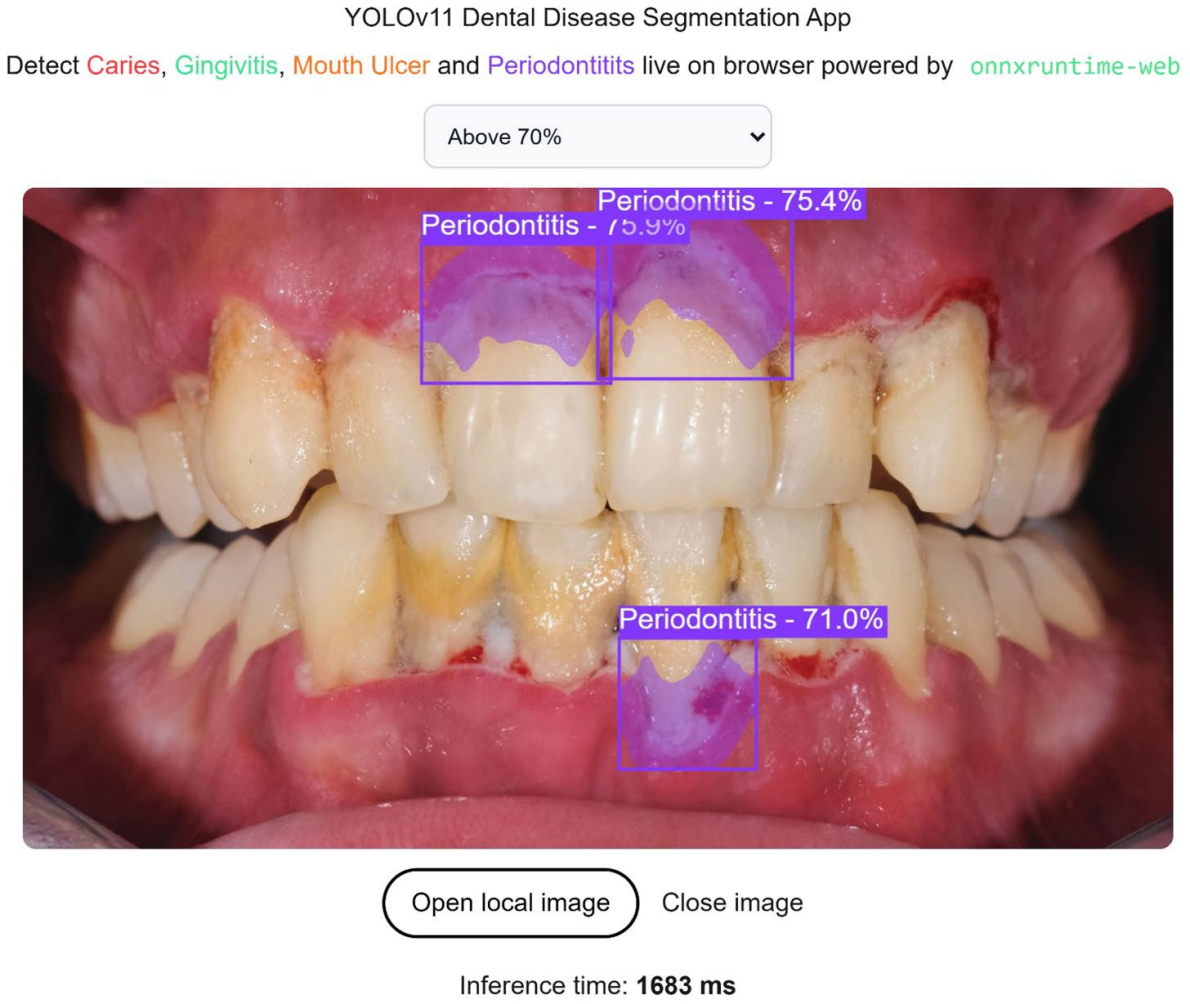
Inference result of web application.

**FIGURE 6:**
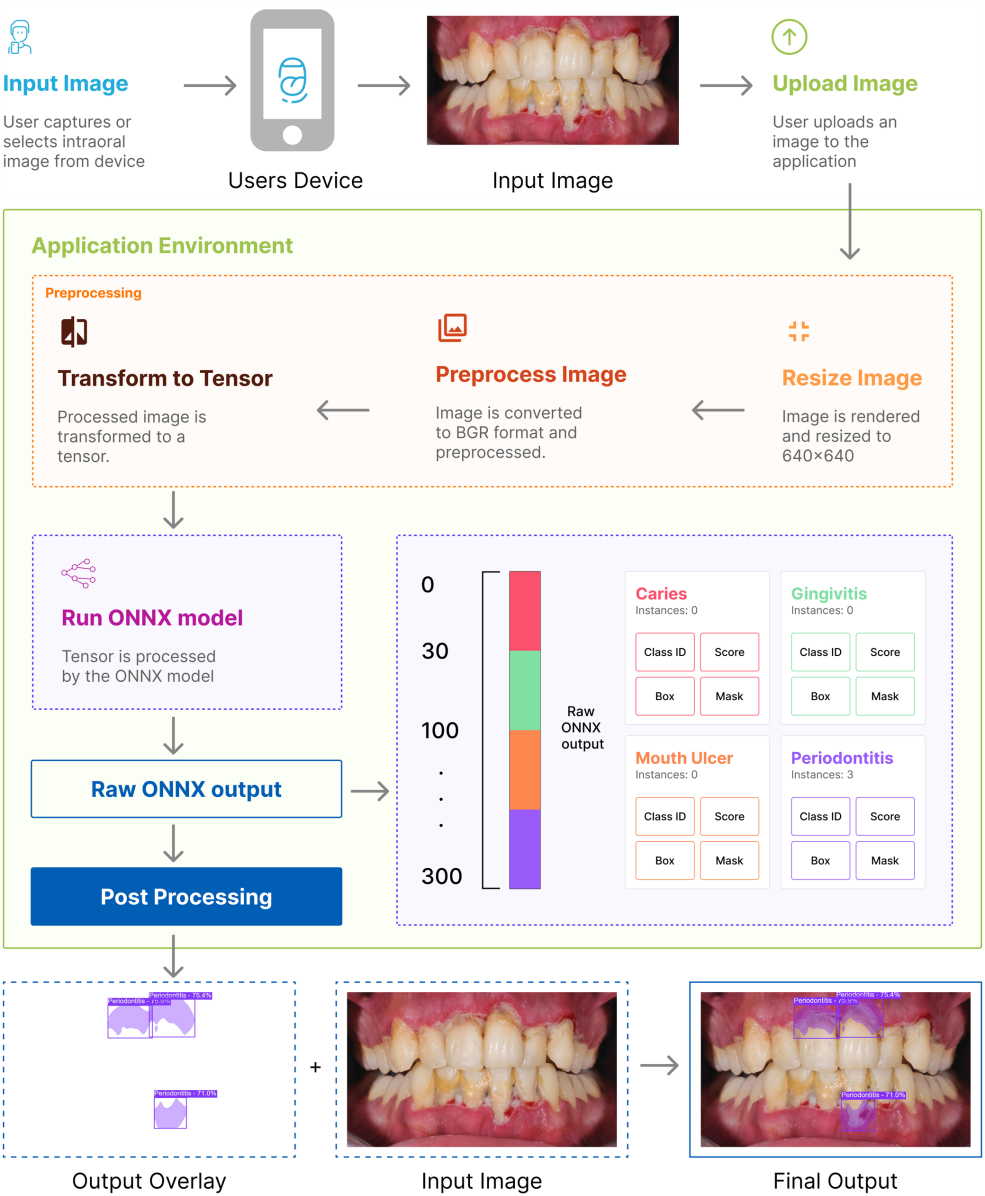
Image detection cycle of the application.

In our web‐based demonstration (Figure 5), users begin by uploading an intraoral photograph, which is immediately rendered and resized to 640 × 640 pixels on an HTML5 canvas. Within the browser all powered by React.js and ONNX Runtime Web the image undergoes a series of preprocessing steps via OpenCV.js: first, it is converted from RGBA to BGR format and normalized, then encapsulated as a blob tensor. This tensor is passed right into the ONNX‐ exported YOLOv11‐seg model, which runs fully client‐side using WebAssembly or WebGPU for quick, platform‐agnostic inference. The model gives two raw outputs: a 300 × 38 array with bounding box coordinates, confidence scores, class indices, and mask‐coefficient vectors; and a 32 × 160 × 160 mask prototype tensor. In JavaScript, we apply confidence‐threshold filtering and class sanitization before reconstructing each instance's segmentation mask by linearly combining the mask coefficients with the prototype masks, applying a sigmoid activation, cropping to the predicted box region, and then up sampling to the bounding‐box size using cubic interpolation (INTER_CUBIC) a method that fits a smooth, third‐degree polynomial through each set of four nearest pixel values to produce a higher‐quality, visually accurate enlargement. After interpolation, we threshold the resulting mask at 0.5 to obtain a binary mask and map it back to the original image space. Box coordinates are then floored, ceiled, and clamped to the original image dimensions. As illustrated in Figure 6, we finally draw each detection's colored bounding box and label, overlay its semi‐transparent binary mask (*α* = 0.4), and render the annotated image to the canvas. This fully client‐side pipeline delivers low latency (∼1600–1900 ms per image), privacy‐preserving, real‐time segmentation of caries, gingivitis, mouth ulcers, and periodontitis requiring no external servers or APIs and offering clinicians a one‐click diagnostic tool directly in any modern web browser.

### 4.2 YOLOv11n‐seg Results

Each phase of training based on a distinct variant of the model has been described in Table 6.

**TABLE 6:** Result of YOLOv11n‐seg for each class across phases of training.

Phase	Class	Box precision	Box recall	Box mAP@50	Box mAP@50–95	Mask precision	Mask recall	Mask mAP@50	Mask mAP@50–95
FE	All	0.519	0.564	0.483	0.263	0.511	0.541	0.474	0.226
	Caries	0.398	0.565	0.426	0.169	0.371	0.522	0.403	0.166
	Gingivitis	0.551	0.321	0.470	0.275	0.548	0.317	0.455	0.212
	Mouth ulcer	0.822	0.565	0.623	0.393	0.824	0.565	0.634	0.367
	Periodontitis	0.305	0.804	0.414	0.214	0.300	0.761	0.403	0.157
PFT	All	0.549	0.535	0.523	0.290	0.544	0.529	0.510	0.246
	Caries	0.652	0.530	0.561	0.270	0.652	0.530	0.555	0.243
	Gingivitis	0.452	0.565	0.463	0.274	0.434	0.543	0.432	0.207
	Mouth ulcer	0.746	0.522	0.602	0.381	0.746	0.522	0.590	0.348
	Periodontitis	0.345	0.522	0.467	0.236	0.345	0.522	0.461	0.188
FFT	All	0.519	0.564	0.483	0.263	0.511	0.541	0.474	0.226
	Caries	0.398	0.565	0.426	0.169	0.371	0.522	0.403	0.166
	Gingivitis	0.551	0.321	0.470	0.275	0.548	0.317	0.455	0.212
	Mouth ulcer	0.822	0.565	0.623	0.393	0.824	0.565	0.634	0.367
	Periodontitis	0.305	0.804	0.414	0.214	0.300	0.761	0.403	0.157

**FIGURE 7:**
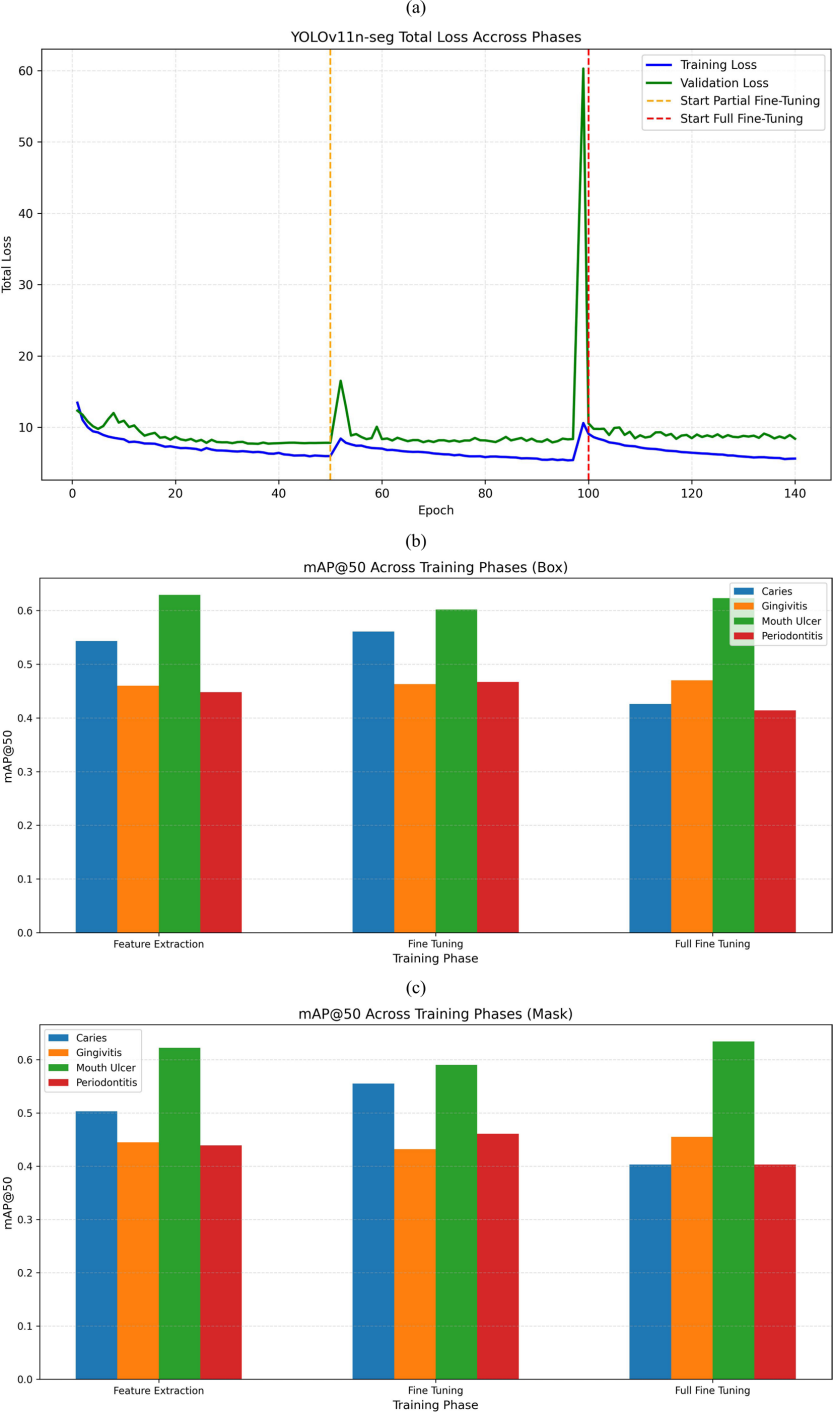
Results of YOLOv11n‐seg model. (a) Train and validation loss, sudden spike indicated model warmup after resuming training from last phase, (b) mAP@50 score of Box, (c) mAP@50 score of Mask.

Figure 7 shows the complete training path of the YOLOv11n‐seg model over three progressive steps: Feature Extraction, partial fine tuning, and full fine‐tuning spanning 138 out of a planned 350 epochs. Throughout Feature Extraction (0–50 epochs), both training and validation losses take a nice smooth curve gradually decreasing. By the end of this phase, the total training loss gets down to about 6 and validation loss settles around 8. At this point in the model, we get a decent box mAP@50 of 0.48 and mask mAP@50 of 0.47 which signifies successful early learning of generalized features. At epoch 51, indicated by the orange vertical line, partial fine tuning starts with more model layers unfrozen. A short increase in validation loss shows right after this change is a typical effect occurs during model “warm‐up” as the network readjusts to new gradients. This is very soon followed by stabilization and further loss reduction. Performance gets much better in this phase, with box mAP@50 going up to 0.523 and mask mAP@50 to 0.51 the top scores seen across all stages. Another sharp increase in validation loss at epoch 100, indicated by the change to full fine tuning (red vertical line) where every layer of the model becomes trainable. Just like the earlier phase change, this spike represents the re‐initialization and adaptation of the deeper layers. Despite keeping up with more epochs through 138, the model does not get better than its earlier best performance. Actually, the mAP values start to go back a little toward the levels seen at the end of feature extraction. In general, the training curve has distinct phase transitions with a general downward trend in losses which validates the structured, staged approach. The spikes at phase changes are showing expected model readjustments. Most importantly, partial fine tuning proves to be the most effective it provides a better trade‐off between learning capacity and stability and ultimately produces highest performance in both detection and segmentation tasks.

### 4.3 YOLOv11s‐Seg (Table 7)

**TABLE 7:** Result of YOLOv11s‐seg for each class across phases of training.

Phase	Class	Box (P)	Box (R)	Box mAP@50	Box mAP@50–95	Mask (P)	Mask (R)	Mask mAP@50	Mask mAP@50–95
FE	All	0.479	0.587	0.487	0.261	0.474	0.581	0.481	0.221
	Caries	0.624	0.543	0.567	0.216	0.646	0.565	0.590	0.212
	Gingivitis	0.392	0.435	0.395	0.209	0.365	0.412	0.355	0.136
	Mouth ulcer	0.642	0.543	0.554	0.396	0.637	0.543	0.552	0.377
	Periodontitis	0.258	0.826	0.433	0.225	0.250	0.804	0.425	0.160
PFT	All	0.440	0.609	0.493	0.282	0.565	0.484	0.490	0.243
	Caries	0.532	0.543	0.501	0.211	0.687	0.478	0.513	0.208
	Gingivitis	0.349	0.609	0.482	0.297	0.415	0.348	0.453	0.213
	Mouth ulcer	0.592	0.543	0.581	0.399	0.836	0.565	0.615	0.377
	Periodontitis	0.288	0.739	0.408	0.222	0.323	0.543	0.380	0.172
FFT	All	0.491	0.543	0.488	0.258	0.550	0.489	0.481	0.221
	Caries	0.550	0.425	0.472	0.193	0.641	0.370	0.479	0.186
	Gingivitis	0.433	0.630	0.460	0.272	0.444	0.543	0.440	0.203
	Mouth ulcer	0.652	0.531	0.587	0.354	0.779	0.543	0.595	0.327
	Periodontitis	0.329	0.587	0.433	0.212	0.336	0.500	0.410	0.169

**FIGURE 8:**
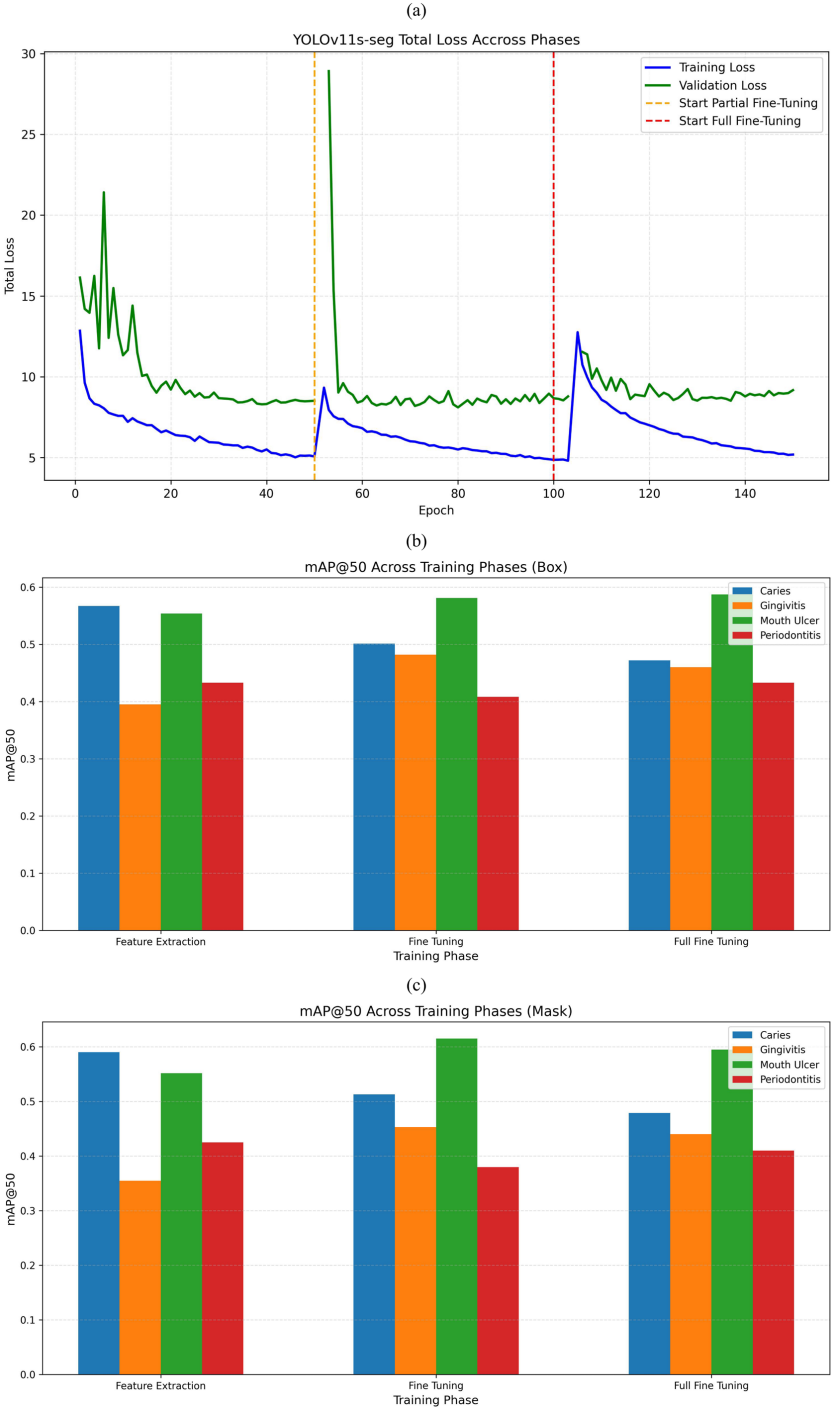
Results of YOLOv11s‐seg model. (a) Train and validation loss, sudden spike indicated model warmup after resuming training from last phase, (b) mAP@50 score of Box, (c) mAP@50 score of Mask.

Figure 8 shows the total loss curves for the YOLOv11s‐seg model in all three phases of training: Feature extraction (FE), partial fine tuning (PFT), and full fine tuning (FFT). In the Feature Extraction phase (1–50 epochs) both training and validation losses decrease gradually from around 12 to 7; Training Loss from around 16 to 9 Validation Loss showing solid initial learning and making good use of available pretrained features. At epoch 50, marked by the orange dashed line, partial fine tuning begins. As expected, there's a sharp spike in validation loss (up to ~29) as newly unfrozen layers begin to adjust under a lower learning rate a typical “warm‐up” effect. The model recovers very quickly, and the losses get stabilized and continue to drop around 5.5 (train) and 8.5 (validation) by epoch 100. During this phase, best performance is achieved by the model with box mAP@50 = 0.493 and mask mAP@50 = 0.490 for “all” class. At the start of full fine tuning (red dashed line at epoch 100), a second validation loss spike is observed, again reflecting the re‐adaptation of fully unfrozen layers. While losses gradually decrease again ending near 5.0 (train) and 8.7 (validation) by epoch 150 the performance gains plateau. Final mAP scores take a small dip from PFT, with box mAP@50 finishing at 0.488 and mask mAP@50 at 0.481. To summarize, the Feature Extraction phase gave us a good baseline, and partial fine tuning was the most impactful because it gave us the best detection and segmentation performance. Full fine tuning proved that going further in unfreezing does not always help. The loss spikes at each phase transition are totally expected they reflect the model's natural adjustment to layers that it can now train.

### 4.4 YOLOv11m‐Seg (Table 8)

**TABLE 8:** Result of YOLOv11m‐seg for each class across phases of training.

Phase	Class	Box (P)	Box (R)	Box mAP@50	Box mAP@50–95	Mask (P)	Mask (R)	Mask mAP@50	Mask mAP@50–95
FE	All	0.491	0.462	0.435	0.256	0.501	0.466	0.445	0.224
	Caries	0.513	0.370	0.406	0.194	0.543	0.387	0.453	0.184
	Gingivitis	0.370	0.500	0.423	0.238	0.361	0.478	0.405	0.178
	Mouth ulcer	0.769	0.522	0.608	0.420	0.774	0.522	0.608	0.392
	Periodontitis	0.311	0.457	0.303	0.171	0.327	0.478	0.312	0.141
PFT	All	0.574	0.566	0.521	0.277	0.560	0.553	0.500	0.228
	Caries	0.420	0.522	0.418	0.163	0.401	0.500	0.388	0.150
	Gingivitis	0.445	0.674	0.470	0.268	0.410	0.630	0.419	0.183
	Mouth ulcer	0.883	0.491	0.630	0.395	0.883	0.494	0.627	0.370
	Periodontitis	0.547	0.578	0.567	0.284	0.546	0.587	0.568	0.209
FFT	All	0.545	0.451	0.472	0.246	0.541	0.429	0.453	0.187
	Caries	0.704	0.217	0.417	0.151	0.674	0.196	0.369	0.122
	Gingivitis	0.411	0.435	0.470	0.275	0.413	0.413	0.439	0.165
	Mouth ulcer	0.762	0.587	0.605	0.352	0.781	0.587	0.596	0.323
	Periodontitis	0.304	0.565	0.397	0.205	0.298	0.522	0.406	0.137

**FIGURE 9:**
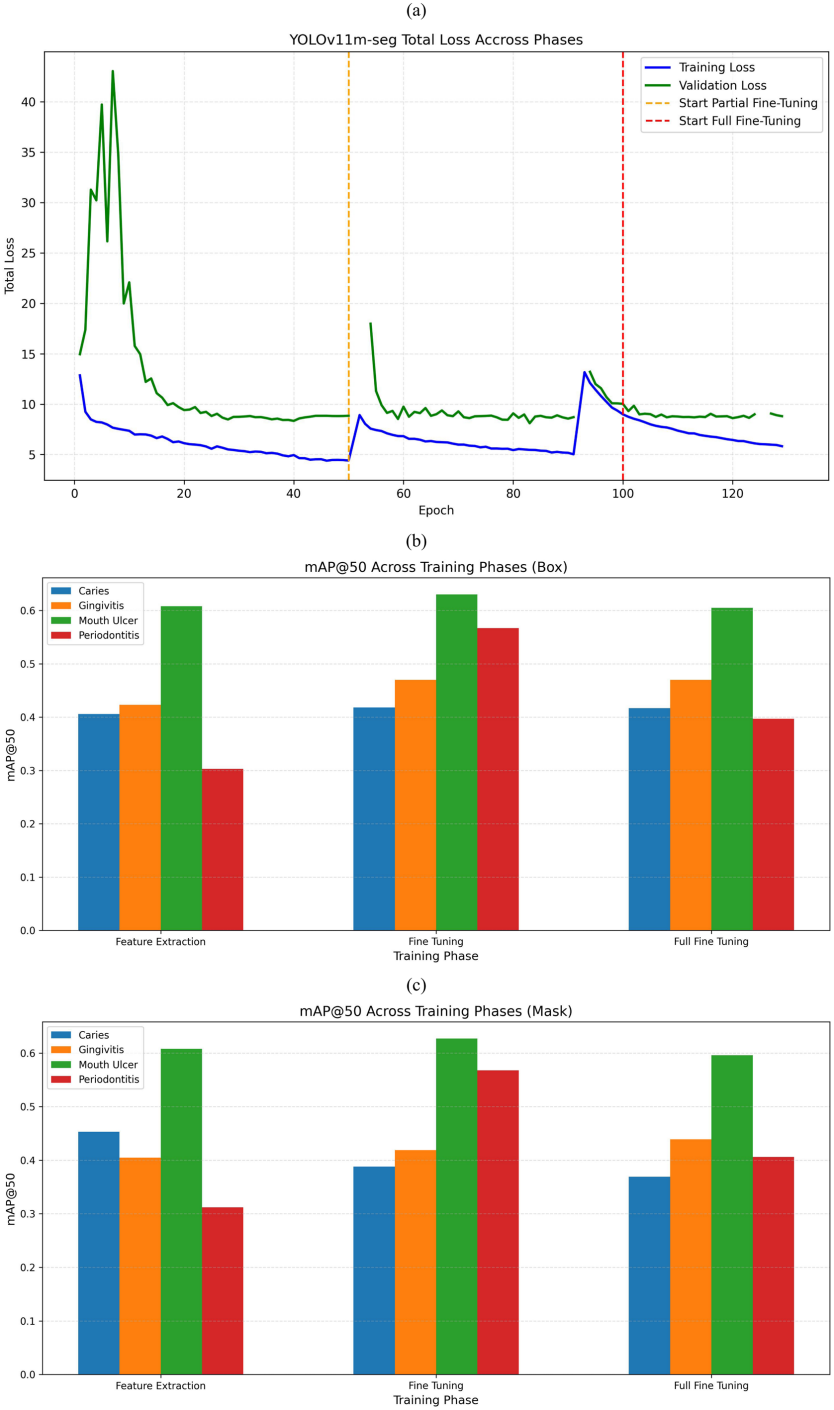
Results of YOLOv11s‐seg model. (a) Train and validation loss, sudden spike indicated model warmup after resuming training from last phase, (b) mAP@50 score of Box, (c) mAP@50 score of Mask.

Figure 9 shows the total training (blue) and validation (green) loss curves for the YOLOv11m‐seg model over all three phases of training: Feature extraction (FE), partial fine tuning (PFT), and full fine tuning (FFT). In the feature extraction phase (epochs 1–50), the training and validation losses both decrease fairly smoothly from around 13 to about 4.5 for training loss and from around 15 to about 8.5 for validation loss indicating good initial learning from the pretrained backbone. At epoch 50 (marked by the orange dashed line), Partial Fine Tuning begins, with additional layers unfrozen. As expected, there's a sharp spike in both loss curves especially validation loss, which briefly jumps to around 18 a typical “warm‐up” effect as the model adjusts to the newly trainable layers. However, this is short lived: losses quickly resume their downward trend, reaching about 5.5 (train) and 8.5 (validation) by epoch 100. This phase also achieves the highest detection accuracy, with box mAP@50 = 0.521 and mask mAP@50 = 0.500 for the “all” class. At epoch 100 (red dashed line) full fine‐tuning starts, unfreezing the whole network. A little spike happens again showing how the model changes with more complex training. While losses keep going down settling near 5.8 (train) and 9.0 (validation) by epoch 135 the performance does not beat the best it got during PFT. Final scores fall a bit with box mAP@50 = 0.472 and mask mAP@50 = 0.453. In short, feature extraction gives a firm base, partial fine‐tuning shares the best balance between learning stability and performance, and full fine tuning proves that more unfreezing can result in lowering returns. The short loss jumps at every phase change are awaited as the model tweaks to new trainable parts.

## 5 Discussion

Experiments conducted on YOLOv11n‐seg, YOLOv11s‐seg, and YOLOv11m‐seg models revealed a definite pattern: results in the partial fine‐tuning phase are results to be expected with YOLOv11m‐seg to be best. It achieved a box mAP@50 of 0.521, precision of 0.574, recall of 0.566 for object localization and mask mAP@50 of 0.500, precision of 0.560, recall of 0.553 for object segmentation both better than feature extraction and full fine‐tuning phases. This aligns with previous dental AI work showing that moderately complex architectures plus selective fine tuning do better than fully re‐trained networks when data are limited (Fatima et al. 2023). The performance difference between the nano and medium versions shows the need for enough representational capacity. While YOLOv11n‐seg is very efficient, its shallow depth limits its ability to capture subtle lesion features. YOLOv11s‐seg gets better on this trade‐off but still is not as good as YOLOv11m‐seg. These outcomes mirror findings on MobileNetV2 and EfficientNet backbones, which demonstrate that somewhat deeper CNNs better represent fine dental pathologies (Fatima et al. 2023). They also echo the gains reported by Hua et al. (2025) in YOLO‐DentSeg, where attention modules improved gingival and carious lesion segmentation. We deliberately omitted larger YOLOv11 variants (l and x) because their much greater parameter counts risk overfitting on a moderately sized dataset—a concern well documented in small scale medical fine‐tuning studies (Fatima et al. 2023; Ramírez‐Pedraza et al. 2025). Instead, we used a balanced annotation strategy that equalized labeled instances per disease class rather than just balancing image counts. This helped the model learn rarer or subtler conditions like early gingivitis and mild periodontitis (Zhang et al. 2023; Wen et al. 2024). Clinically, our work advances the field by detecting four distinct oral diseases: caries, gingivitis, mouth ulcers, and periodontitis from simple intraoral photographs. This scope is broader than many prior studies, which either focused on a single condition (Park et al. 2022; Tareq et al. 2023) or required radiographic inputs (Zhu et al. 2023; Chen et al. 2023). Our unified, single pass YOLOv11‐seg pipeline handles both detection and segmentation in one step, matching or exceeding the performance of more complex multi‐stage approaches like Faster R‐CNN or U‐Net (Park et al. 2022, 2023). To the best of our knowledge this study is the first to present a comprehensive, multi‐phase evaluation of YOLOv11‐Seg variants for multi‐condition intraoral disease segmentation, introducing a fully client‐side ONNX deployment pipeline for low resource real‐time clinical screening enabling both clinicians and non‐experts to detect oral diseases from intraoral images with a single click. A key strength of this study is its emphasis on real world deployment. We exported our best model to ONNX (with built‐in NMS) and integrated it into a React.js web application via ONNX Runtime Web. The result is a fully client‐side, browser‐based inference system that runs in real time, needs no server infrastructure, and preserves user privacy since data never leaves the user's device. This setup is especially suitable for resource constrained or intermittently connected environments—an important trend in AI for healthcare (Cheng et al. 2024; Hua et al. 2025; Ramírez‐Pedraza et al. 2025). Some limitations still persist. The initial dataset of 582 high quality manually annotated images is on the smaller side and is not rich in demographic and environmental conditions. While augmentation enabled expanded v2 and v3 datasets to be created, these synthetic upgrades cannot fully substitute for the variability that real world clinical settings bring. Model training happened on Google Colab's free‐tier (Intel Xeon CPU, 13 GB RAM, T4 GPU, 120 GB storage) which means there was little room for trying bigger models and just small changes in hyperparameters. How well this model does when things get tricky—like with blockages, bad light, or when images are blurry from movement—has not been tested in real world clinical workflows. In spite of these limitations, the system showed very good training convergence, strong per class segmentation, and an easy‐to‐use interface. The minimalist, “one‐click” UI that needs just an image upload to give instant, color coded segmentation overlays is designed keeping in mind both dental professionals as well as general users. This approach aligns with the best practices of user experience in mobile health applications available to everyone without compromising diagnostic utility.

## 6 Conclusion

To conclude, the study proves that a moderately sized, efficiently fine‐tuned segmentation model such as YOLOv11m‐seg is capable of providing precise real‐time detection for a variety of dental conditions. The choices we made with regard to model selection, balanced annotations, and browser‐based deployment have broken down barriers to early disease screening wherever there is a clinical setting or limited resources available. Model performance was validated through distinct training, validation, and test splits; although k‐fold cross‐validation was considered, it was not implemented due to computational constraints associated with using Google Colab's free tier. Future work will include expansion to broader pathologies and k‐fold cross‐validation to further quantify robustness. The findings give more weight to the idea of implementation of deep learning in dental diagnostics and it also adds another little push to this huge wave in AI‐powered oral health monitoring (Fatima et al. 2023; Park et al. 2022; Hua et al. 2025; Ramírez‐Pedraza et al. 2025).

## Author Contributions

**Pranta Barua:** conceptualization, investigation, writing – original draft, methodology, validation, writing – review and editing, software, formal analysis, data curation, resources. **Md Rakibul Islam:** conceptualization, investigation, writing – original draft, writing – review and editing, validation, methodology, visualization, software, formal analysis, data curation, resources. **Mahmud Uz Zaman:** conceptualization, writing – review and editing, writing – original draft, project administration. **Adel Alenazi:** conceptualization, writing – original draft, writing – review and editing, project administration. **Fawaz Alqahtani:** project administration, writing – review and editing, writing – original draft, conceptualization. **Huda Abutayyem:** conceptualization, writing – original draft, writing – review and editing, project administration, funding acquisition. **Maher Al Shayeb:** project administration, writing – review and editing, writing – original draft, conceptualization, funding acquisition. **Ruba Odeh:** conceptualization, writing – original draft, writing – review and editing, project administration. **Nesrine A. Elsahn:** conceptualization, writing – original draft, writing – review and editing, project administration. **Mohammad Khursheed Alam:** conceptualization, writing – original draft, writing – review and editing, project administration, supervision, resources, funding acquisition, investigation, methodology, validation, visualization, formal analysis, software.

## Ethics Statement

The authors have nothing to report.

## Consent

The authors have nothing to report.

## Conflicts of Interest

The authors declare no conflicts of interest.

## Data Availability

All data generated or analyzed during this study is included within the article (Roboflow Universe 2024, 2025a, 2025b; Jocher et al. 2024; Dwyer et al. 2024).
